# Differences in Androgen Receptor Expression in Human Heart Tissue in Various Types of Cardiomyopathy and in Aortic Valve Stenosis

**DOI:** 10.3390/jcdd10110466

**Published:** 2023-11-17

**Authors:** Katja Eildermann, Sabrina Goldmann, Ulrich Krause, David Backhoff, Friedrich A. Schöndube, Thomas Paul, Thomas Quentin, Matthias J. Müller

**Affiliations:** 1 Department of Pediatric Cardiology and Intensive Care Medicine, Georg-August-University Goettingen, 37077 Goettingen, Germany; katja.eildermann@med.uni-goettingen.de (K.E.); sabrina.goldmann@med.uni-goettingen.de (S.G.); ukrause1@gwdg.de (U.K.); david.backhoff@med.uni-goettingen.de (D.B.); tpaul@gwdg.de (T.P.); 2 Department of Thoracic and Cardiovascular Surgery, Georg-August-University Goettingen, 37077 Goettingen, Germany; friedrich.schoendube@med.uni-goettingen.de; 3 Department of Clinical Pharmacology and Toxicology, University Medical Centre Hamburg Eppendorf, 20246 Hamburg, Germany

**Keywords:** androgen receptor, human heart tissue, cardiomyopathy, aortic valve stenosis

## Abstract

**Background**: Sex-specific differences in heart disease outcomes are influenced by the levels of the steroid hormones, estrogen and testosterone. While the roles of estrogen receptors in cardiac disease are well-studied in animals and humans, respective research on androgen receptors (AR) is limited. Here we investigate AR protein and mRNA expression in human myocardium of various cardiac diseases. **Methods**: AR expression was analyzed by western blotting in myocardium from human non-failing hearts (NF, n = 6) and patients with aortic stenosis (AS, n = 6), hypertrophic cardiomyopathy (HCM, n = 7), dilated cardiomyopathy (DCM, n = 7), and ischemic cardiomyopathy (ICM, n = 7). Using an AR45-specific antibody, a subsequent western blot assessed samples from male and female patients with HCM (n = 10) and DCM (n = 10). The same sample set was probed for full-length *AR* and *AR45* mRNA expression. Immunohistochemistry (IHC) localized AR in myocardium from HCM and AS hearts. **Results**: Full-length AR was notably enriched in AS and HCM hearts compared to ICM, DCM, and NF. Similarly, AR45 was more abundant in HCM than in DCM. In contrast to the pattern observed for AR protein, full-length *AR* mRNA levels were lower in HCM compared to DCM, with no discernible difference for the *AR45* isoform. Although gender differences in AR expression were not detected in western blots or qRT-PCR, IHC showed stronger nuclear AR signals in males than in females. **Conclusions**: Our findings indicate disease-specific regulation of *AR* mRNA and/or AR protein in cardiac hypertrophy, underscoring a potential role in this cardiac pathology.

## 1. Introduction

Sex-specific differences have been identified in adult patients with valvular aortic stenosis (AS) and left ventricular hypertrophic cardiomyopathy (HCM). Men, in comparison to age-matched women, appear to be more susceptible to HCM, with distinct variations in heart size [1,2].

The gender-related disparities in disease outcomes are hypothesized to arise from the differential levels of sexual steroids, notably estrogen and testosterone [3,4]. While the exact hormonal mechanisms remain elusive, estrogens are postulated to confer protection against hypertrophic responses, whereas androgens are believed to promote them [1].

While estrogen predominantly interacts with estrogen receptors α and β (ERα, ERβ), testosterone associates with the androgen receptor (AR) [5]. Steroid receptors, functioning as ligand-activated transcription factors, bind to specific DNA response elements, thereby influencing a variety of biological processes [6]. In gender-related cardiac research, the most extensively studied sex steroid receptors are ERα and ERβ, both identified in human cardiac tissue at both mRNA and protein levels [7]. Furthermore, heightened protein expression of ERs has been recognized in myocardium from AS [7] and dilated cardiomyopathy (DCM) patients [8].

In contrast, the cardiac role of AR is relatively understudied. Yet, its presence in cardiac myocytes across different species, including humans, is well-documented [9]. An AR-driven hypertrophic response prompted by testosterone has been identified in vitro in cardiomyocytes [9].

Interestingly, the heart not only expresses full-length AR but also a heart specific AR isoform called AR45 [6]. AR45 is composed of a unique N-terminal extension, the Exon 1B, which is conserved across the genomes of various Old and New World monkeys, dogs, pigs, and elephants [10]. Exon 1B is linked to the DNA-binding domain, hinge region, and ligand-binding domain of the AR [6]. AR45 is known to bind androgen and localizes to the cell nucleus [6]. Interestingly, AR45 has been shown to inhibit AR function by forming AR-AR45 heterodimers [6]. Its restricted expression pattern, the inhibitory effect on AR function, and the cofactor-dependent activity suggest an important biological role of AR45 in modulating androgen action. However, the specific functions of AR45 in the heart have not been extensively studied.

In this study, we assess AR protein expression in various cardiac diseases using western blotting. We analyze myocardial samples from HCM and DCM patients, focusing on gender-specific differences in AR45 protein and full-length *AR* and *AR45* mRNA expression. Lastly, we study AR protein localization in HCM and AS myocardium using immunohistochemistry.

## 2. Methods

### 2.1. Patient Material

Procedures for obtaining human tissue were in accordance with the principles outlined in the Declaration of Helsinki. The respective local Ethics Committees of the University of Hamburg and the University of Goettingen in Germany granted permission for the use of this human material (Az. 532/116/9.7.1991).

To analyze full length AR protein expression by Western blotting we used six nonfailing (NF) human hearts (2 females, mean age: 44 years; 4 males, mean age: 50 years) that were obtained from prospective organ donors that could not be transplanted due to technical reasons. Seven DCM samples (1 female, mean age: 46 years; 6 males, mean age: 41 years) and seven ICM samples (7 males, mean age: 57 years) were taken from patients undergoing heart transplantation due to end-stage heart failure. NF, DCM, and ICM samples have already been described in studies published by Stein et al. and El-Armouche et al. [11,12] and were taken from the left ventricle. Six samples from patients with AS (4 females, mean age: 72 years; 2 males, mean age: 75 years) and seven HCM samples (n = 7; 3 females, mean age: 64 years; 4 males, mean age: 65 years) were taken from the upper left outflow tract below the aortic valve during surgical repair of left outflow tract obstruction. 

For AR45 Western blot and qRT-PCR analysis of AR45 and full-length AR, a second set of tissue was implemented consisting of 10 HCM samples (5 female, mean age: 63 years; 5 male, mean age 47 years) and 10 DCM samples (5 female, mean age: 41 years; 5 male, mean age: 49 years). For IHC, we used 10 HCM samples (5 female, 5 male) and 10 AS samples (5 female, 5 male).

Freshly taken tissue samples were snap-frozen in liquid nitrogen and then stored at −80 °C or fixed in Formalin.

### 2.2. Protein Isolation, SDS-PAGE, Western Blotting, and Statistical Analysis

Frozen tissue powder from tissue samples of NF, DCM, ICM, AS, and HCM patients, which were used for full-length AR western blotting was dissolved in lysis buffer (20 mM Tris, pH 7.4, 50 mM NaCl, 50 mM NaF, 5 mM Na-Pyrophosphat, 5 mM sucrose, 10 mM DTT, 1% Triton X-100 (*v*/*v*), complete protease inhibitors and PhosSTOP^TM^ phosphatase inhibitors (Roche, Basel, Switzerland)) and further processed by sonication. A total of 35 µg tissue protein extract per lane was subjected to SDS-PAGE (10% polyacrylamide, Roth, Karlsruhe, Germany). From a second set of samples, our objective was to extract both protein and mRNA. We used frozen tissue powder from the myocardium of twenty patients: ten with HCM (five males and five females) and ten with DCM (five males and five females). This powder was dissolved in Trizol® reagent (Peqlab, Erlangen, Germany). Following the manufacturer’s instructions, we isolated protein alongside mRNA. The resultant pellets were dissolved in lysis buffer and processed further by sonication. We subjected 15 µg of the tissue protein extract per lane to SDS-PAGE. Size-separated protein was transferred from the SDS-PAGE into a nitrocellulose membrane (Schleicher & Schuell BioScience, Inc., Keene, NH, USA) by wet blotting. The membrane was then blocked in TBS buffer pH 7.6 containing 5% milk powder for 2 h at room temperature. Incubation with the primary antibody was performed overnight at 4 °C in TBS buffer containing 1% Tween and 5% milk powder. Monoclonal rabbit anti-androgen receptor (clone EPR1535, Epitomics, Burlingame, CA, USA) was used at a dilution of 1:1000 to detect full-length AR at 110 kDa. EPR1535 detects an N-terminal epitope on Exon 1. The monoclonal rabbit anti-androgen receptor (clone SP242, Spring Bioscience, Pleasanton, CA, USA) was used at a dilution of 1:300 to detect AR isoform 45 at 45 kDa. SP242 detects a c-terminal epitope. AR receptor signals were normalized to GAPDH expression, that was determined using the monoclonal rabbit anti-GAPDH antibody, clone 14C10 (Cell Signaling, Pleasanton, CA, USA) at a dilution of 1:20,000. HRP-conjugated secondary antibodies were diluted in TBS buffer containing 1% Tween and 5% milk powder for 1 h at room temperature. Chemiluminescence was detected using SuperSignal^TM^ West Femto reagent (Thermo Fisher Scientific, Waltham, MA, USA) and an LAS-3000 Imaging System (Fujifilm, Minato, Tokyo, Japan).

To detect and compare the full-length AR, we loaded each blot with a sequence of NF, DCM, ICM, AS, and HCM samples. To ensure consistent comparisons across different blots, we used a calibration sample made up of a protein lysate from the left ventricular myocardium of another explanted heart. This was loaded onto both the left and right external lanes of each Western blot. For the analysis of AR 45 in the HCM and DCM samples, we followed a similar approach, sequentially loading samples from HCM males, HCM females, DCM males, and DCM females, flanked by the calibration sample.

We quantified the signal intensities from the specific protein bands densitometrically. The calibration sample's signal intensities were established as the 100% baseline, and we then related the signal intensities of each sample to this calibration sample. Additionally, all samples were normalized to the housekeeping protein, GAPDH. To determine statistical significance, we set a threshold at p < 0.05 and utilized ANOVA followed by Tukey’s posttest or the unpaired *t*-test.

### 2.3. mRNA Isolation and qRT-PCR

For mRNA isolation from HCM and DCM, we utilized the same Trizol^TM^ (Peqlab, Erlangen, Germany) lysate as we did for protein isolation. We extracted the total RNA following the manufacturer’s instructions. Subsequently, we purified the resulting RNA using the RNeasy® Mini Kit (Qiagen, Hilden, Germany). Reverse transcription was carried out with 1 µg total RNA, the Superscript II Reverse Transcriptase (Invitrogen, Carlsbad, CA, USA), and random hexamer primers (Invitrogen, Van Allen Way Carlsbad, CA, USA). 

To distinguish between full-length AR and AR45, we designed isoform-specific primers. For both PCRs, we utilized the same reverse primer that binds to AR exon 3: 5′-GAAGACCTTGCAGCTTCCAC-3′. However, the forward primers were tailored to specifically target each isoform. For the detection of full-length AR, we employed a forward primer that binds to AR exon 1A: 5′-GGTGAGCAGAGTGCCCTATC-3′. This exon is present in full-length AR but absent in AR45. Conversely, to detect the AR45 isoform, we utilized a forward primer that targets the AR45-specific exon 1B: 5′-GGTGAGCAGAGTGCCCTATC-3′. This primer does not bind to full-length AR. 

We normalized AR expression to GAPDH, using the forward primer 5′-GAGTCAACGGATTTGGTCGT-3′ and the reverse primer 5′-GACAAGCTTCCCGTTCTCAG-3′. The qPCR was executed using the iQ SYBR® Green Supermix (BioRad, Hercules, CA, USA) on the MyiQTM RT-PCR Detection System (BioRad, Hercules, CA, USA). For data evaluation, we utilized the iQTM5 software from BioRad (Version 2.1).

### 2.4. Immunohistological Staining of AR in Cardiac Disease

Formalin-fixed myocardial tissue from AS (n = 10; 5 male; 5 female), and HCM (n = 10; 5 male, 5 female) hearts were embedded in paraffin and then sectioned into 5 µm thick slices, ready for immunohistological staining following standard protocols. The tissue slices underwent deparaffinization, and antigens were retrieved using a high pH target retrieval buffer (GV804) from DAKO (Santa Clara, CA, USA), with a 20-min incubation at high pressure and 90 °C. Subsequently, the primary antibodies were incubated overnight. To detect AR in cardiac tissue, we utilized two antibodies: A rabbit polyclonal Anti-AR antibody that targets the N-terminus of the AR protein (N-20, sc-816) sourced from Santa Cruz (Dallas, TX, USA), used at a 1:300 dilution and a monoclonal rabbit anti-androgen receptor (clone SP242) from Spring Bioscience (Pleasanton, CA, USA) that targets the C-terminus of AR, used at a 1:40 dilution. To enhance the AR signals, we employed the Zytochem Plus HRP Polymer anti-rabbit System (ZUC032-006) from Zytomed (Berlin, Germany), which was visualized using DAB (3,3′-Diaminobenzidine). 

To clearly show the identity of the AR positive cells, we employed a double-staining procedure using the monoclonal rabbit anti-androgen receptor (clone EPR1535) from Epitomics (Burlingame, CA, USA) at a 1:50 dilution with either Vimentin or Desmin as a second primary antibody. Vimentin was detected using a monoclonal mouse anti-Vimentin antibody (IgG1, Clone V9, #M0725) from DAKO (Santa Clara, CA, USA) at a 1:1000 dilution. Desmin detection used the monoclonal mouse anti-Desmin antibody (IgG1, D9, # 10519) from Progen Biotechnik GmbH (Heidelberg, Germany) at a 1:1500 dilution. These signals were amplified using the Histofine Simple Stain AP (Multi) Anti-Mouse and Anti-Rabbit from Medac (Wedel, Germany) and visualized with permanent red.

All immunohistochemical stainings were further processed with Hemalaun to visualize the nuclei in lilac. Controls were implemented using the appropriate IgGs at the relevant concentrations in place of the primary antibody.

## 3. Results

### 3.1. Full-Length AR Protein Expression in AS and HCM Is 3 to 4-Fold Higher Compared to NF, DCM, and ICM Samples

**Figure 1:**
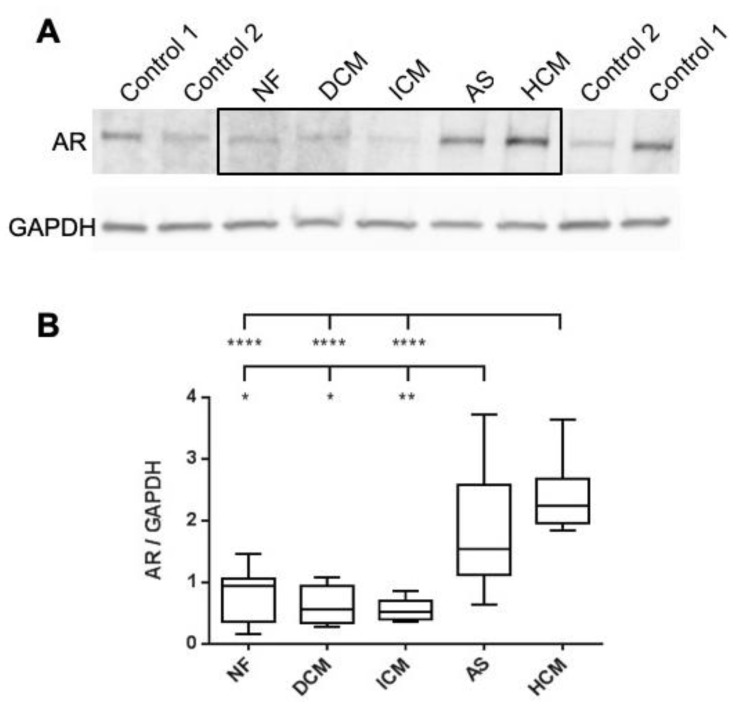
**Western Blot Analysis of AR Protein Levels in Various Cardiac Conditions using the AR-Antibody EPR1535.** (**A**) Representative Western blot demonstrating full-length AR detection at 110 kDa and GAPDH detection at 37 kDa. (**B**) Box and whisker plot illustrating the summarized statistics of normalized AR signals for each cardiac condition. Sample details are as follows: NF (n = 6), DCM (n = 7), ICM (n = 7), AS (n = 6), HCM (n = 7). Disease group comparisons were conducted using the unpaired t-test. Groups with significant variations in AR expression are marked with respective p-values (* = p < 0.05, ** = p < 0.01, **** = p < 0.0001). **Abbreviations**: AR = Androgen receptor; AS = Valvular aortic stenosis; GAPDH = Glyceraldehyde-3-phosphate dehydrogenase (housekeeping gene); NF = Nonfailing; DCM = Dilated cardiomyopathy; ICM = Ischemic cardiomyopathy; HCM = Hypertrophic cardiomyopathy.

AR was detected by western blotting using the antibody clone EPR1535, which binds to the N-terminal region of AR. This enables the detection of the full-length AR at 110 kDa, but not the heart-specific isoform AR45. Total protein from NF (n = 6), DCM (n = 7), ICM (n = 7), AS (n = 6), HCM (n = 7) heart tissues was analyzed. For normalization, a consistent reference sample set was used to bracket the test samples, and AR signal intensity was normalized against GAPDH. An exemplary western blot is depicted in Figure 1A. The summarized statistics of the normalized AR signal intensities for each heart condition are presented as a box and whiskers plot in Figure 1B. 

Distinct variations in AR protein levels were observed across various cardiac disorders. DCM (0.62 ± 0.12) and ICM (0.54 ± 0.07) samples demonstrated average AR protein levels similar to the control group (NF: 0.79 ± 0.18). In contrast, AS (1.82 ± 0.44) and HCM (2.45 ± 0.23) samples exhibited a significant increase, approximately three to four-fold, compared to NF, DCM and ICM hearts.

### 3.2. HCM Samples Display Significantly Higher AR45 Protein Levels Than DCM Samples

**Figure 2:**
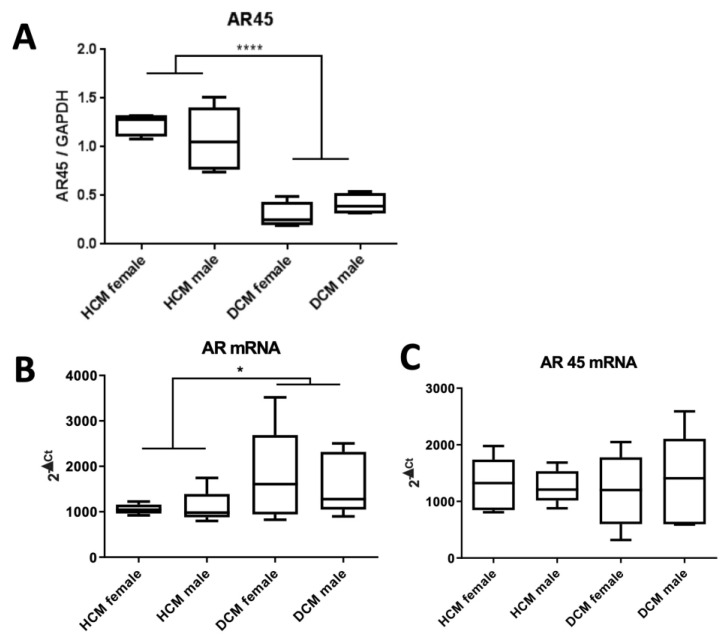
Comparative Analysis of AR Expression in Myocardial Tissues from HCM and DCM Patients comprising n = 5 female and n = 5 male samples: (**A**) Normalized AR45 protein levels, identified through Western blotting using the AR antibody clone SP242. Normalization incorporated a consistent reference sample set bracketing the test samples, with AR signal intensity normalized against GAPDH. (**B**) Quantification of full-length AR mRNA via qRT-PCR, with normalization to GAPDH. (**C**) AR45 mRNA levels were measured using qRT-PCR and normalized to GAPDH. For statistical evaluation, an unpaired t-test was employed. Significance levels are denoted as: * = p < 0.05, **** = p < 0.0001 Abbreviations: AR = Androgen receptor; GAPDH = Housekeeping gene; DCM = Dilated cardiomyopathy; HCM = Hypertrophic cardiomyopathy.

The heart-specific AR isoform, AR45, was detected at 45kDa using the antibody clone SP242. This clone binds to the C-terminal region of AR, allowing for the detection of both the full-length AR and AR45. Total protein from HCM and DCM myocardium, comprising n = 5 female and n = 5 male samples, was analyzed. For normalization, a consistent reference sample set bracketed the test samples, and AR signal intensity was normalized against GAPDH. AR signal intensities for each condition and gender are presented as a box and whiskers plot in Figure 2A. Notably, no differences in signal intensities were observed between males and females. However, HCM samples exhibited significantly elevated levels of AR45 protein compared to DCM samples.

### 3.3. HCM Samples Display Significantly Lower AR mRNA Levels Than DCM Samples, While AR45 mRNA Levels Remain Comparable.

To distinguish between full-length AR and AR45, we used isoform-specific primers on HCM and DCM myocardium, comprising n = 5 female and n = 5 male samples. QRT-PCR analysis showed an increase in full-length AR mRNA in DCM samples compared to HCM samples. Expression levels of AR45 mRNA were similar. No gender-specific differences were detected (Refer to Figure 2B,C).

### 3.4. AR Is Localized in the Nucleus of Cardiomyocytes and Its Presence Is more Pronounced in Male Specimens Than in Females with HCM or AS

**Figure 3:**
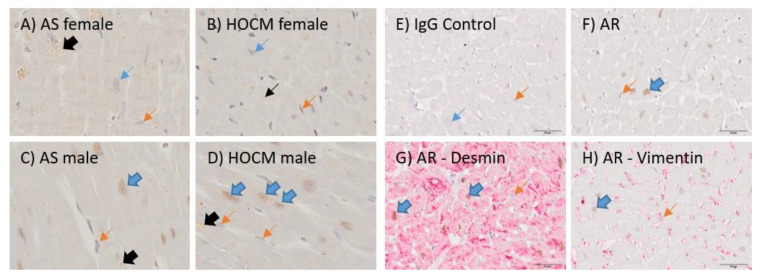
Immunohistochemical Staining of AS and HOCM in Males and Females. (**A**–**D**) Representative AR stainings using the SP242 antibody. Gender and disease type are specified in the figure’s description. (**E**–**H**) Double staining was employed to identify AR-positive cells. AR antibody: EPR1535. Blue arrows indicate cardiomyocytes, orange arrows point to fibroblasts/endothelial cells, and black arrows highlight lipofuscin accumulations. Block arrows indicate a positive staining. Abbreviations: AR = Androgen Receptor; AS = Valvular Aortic Stenosis; HCM = Hypertrophic Cardiomyopathy.

Immunohistochemical single stainings were performed using antibodies sc-816, directed to the N-terminus, and SP242, directed to the C-terminus. These were applied to myocardium from AS (n = 10; 5 male; 5 female) and HCM (n = 10; 5 male, 5 female) hearts. AR signals were observed in the nucleus of cardiomyocytes (Figure 3A–D). Some cytoplasmic signals were presumably unspecific, likely attributed to Lipofuscin.

Contrary to the western blot results, nuclear staining was more pronounced in the myocardium of males compared to females in both HCM and AS specimens. Specifically, only 3 out of 5 female HCM and 1 out of 5 AS patients displayed weak nuclear signals. Conversely, 5 out of 5 male HCM and 3 out of 5 male AS patients showed intense nuclear signals. One male AS patient had a weak signal. Specimens not mentioned did not exhibit any nuclear signal. The results were consistent across both antibodies. 

The identity of the AR-expressing cells was affirmed by their distinct morphological characteristics. This was further validated by the absence of Vimentin and the presence of Desmin, as identified by double staining (Figure 3E–H).

## 4. Discussion

Information on AR in human cardiac tissue [5] is limited. For the first time, we described AR expression in a large cohort of human myocardium.

In our study, both full-length AR and AR isoform 45 were expressed in human myocardium at mRNA and protein levels. AR protein was enriched by 3 to 4-fold in hypertrophied myocardium from AS and HCM patients, compared to NF myocardium or myocardium diagnosed with DCM or ICM. Likewise, the heart-specific isoform AR45 was enriched in HCM compared to DCM, with no significant differences between males and females. Contrarily, AR mRNA levels were reduced in myocardium from HCM patients when compared to that of DCM patients, with no statistically significant difference between males and females and with no difference in the expression of AR45. Our study also described the localization of AR in the nucleus of cardiomyocytes, and in contrast to the western blot results, observed a differential nuclear abundance between male and female hypertrophied cardiomyocytes.

Cardiac hypertrophy acts as a compensatory mechanism in response to increased pressure overload. Myocardial hypertrophy can be triggered by AS [2] due to the heightened resistance of the left outflow tract [13]. Alternatively, in cases of hypertrophic forms of cardiomyopathies, it can be caused by mutations in genes coding for sarcomeric proteins [14].

Sex-specific differences have been identified in adult patients with valvular aortic stenosis (AS) and left ventricular hypertrophic cardiomyopathy (HCM) [1,2]. The cellular and molecular mechanisms underlying sex-specific differences in heart failure remain largely elusive. Differences might be linked to sex-specific variations in gene expression profiles, which are triggered by elevated workload [1]. One potential target gene through which the AR might influence hypertrophic remodeling is TGF-beta. The TGF-beta promoter contains a binding sequence for the AR, which can either upregulate or downregulate TGF-beta expression [15]. In the thoracic aortic constriction (TAC) mouse model, a sex-specific difference in the increase of TGF-beta expression in hypertrophied myocardium, with a more pronounced effect in males than in females, was observed [16]. Epidemiological and experimental studies suggest that AR-specific ligands can induce cardiac hypertrophy. This has been observed in athletes who misuse anabolic steroids [17] and through intramuscular testosterone application in rats [18]. Our findings of enhanced AR abundance in hypertrophied myocardium support this previous research.

Our findings on AR protein expression in the myocardium of AS patients parallel those of Nordmeyer et al., who reported an upregulation of ERα in samples from both female and male AS patients [7]. Contrary to our AR expression data, ERα was also found to be upregulated in DCM patient samples [8].

While no gender-related difference in AR was detected by western blot, IHC showed a differential nuclear abundance in male vs. female hypertrophied myocardium. The findings were consistent with both C- and N-terminal antibodies, indicating that the detected signal stems from full-length AR, or potentially from both isoforms, AR and AR45. Elevated testosterone levels in men [3] might result in a more potent activation of the AR, potentially explaining the divergent clinical trajectories of heart failure between genders. Findings in the mouse, where α1-ARs were located around or within the nucleus but not on the plasma membrane, suggest a potential direct role of ARs in transcriptional regulation, which could influence genes associated with cardiac hypertrophy or other pathological processes [6,19].

Next to the full-length androgen receptor, there exists the variant AR45 that utilizes Exon 1B instead of exon 1A [6]. AR45, predominantly expressed in cardiac muscle [6], showed enhanced levels in hypertrophied myocardium in our Western blot results. AR45 has been demonstrated to play a modulatory role in androgen action within the heart due to its ability to inhibit AR function by forming AR-AR45 heterodimers [6]. Therefore, enhanced AR45 expression in hypertrophied myocardium might be a compensatory effect to inhibit enhanced AR function. However, the exact mechanisms and implications of AR45 in cardiac diseases warrant further investigation.

An intriguing aspect of AR regulation is the contrasting findings of AR mRNA and protein levels in HCM and DCM samples. While protein levels were elevated in HCM, mRNA levels were lower compared to DCM. Similar findings have been observed for vas deferens epithelial cells where *AR* mRNA expression was down-regulated by its ligand, DHT, while the protein levels appeared to be up-regulated under certain conditions [20]. This discrepancy between mRNA and protein levels suggests complex regulatory mechanisms at play, possibly involving post-transcriptional or post-translational modifications.

### 4.1. Limitations

Limitations of the Study and Future Directions: A significant limitation of our research was the small sample size, especially in sex-specific analyses (e.g., only one female DCM sample compared to six male samples), leading to reduced statistical power. Another constraint was that the DCM and ICM patients in our study were in the end-stage of heart failure. Future research should delve deeper into the mechanisms behind AR's role in cardiac hypertrophy, especially focusing on the functional implications of AR45 and the gender-specific effects of AR.

### 4.2. Conclusions

In conclusion, our study marks the first-time demonstration of AR expression at the protein level in a sizable human myocardium cohort. The elevated AR and AR45 protein levels in hypertrophic conditions, coupled with the observed gender differences, highlight the intricate roles of androgen receptors in cardiac diseases. Further research is essential to fully understand these roles and their therapeutic implications.

## Data Availability

Not applicable.
